# First-Principles Study of Bi-Doping Effects in Hg_0.75_Cd_0.25_Te

**DOI:** 10.3390/molecules26164847

**Published:** 2021-08-11

**Authors:** Xueli Sun, Xuejun Su, Dechun Li, Lihua Cao

**Affiliations:** 1 School of Basic Sciences for Aviation, Naval Aviation University, Yantai 264001, China; snow0530@126.com (X.S.); su_xuejun@126.com (X.S.); 2 School of Information Science and Engineering, Shandong University, Qingdao 266200, China; 3 Engineering Training Center, Shandong University, Jinan 250100, China

**Keywords:** first-principles, Hg_0.75_Cd_0.25_Te, Bi doping, electronic structures

## Abstract

First-principles calculations based on density functional theory have been performed for exploring the structural and electronic properties of Bi-doped Hg_0.75_Cd_0.25_Te (MCT), using the state-of-the-art computational method with the Heyd–Scuseria–Ernzerhof (HSE) of hybrid functional to correct the band gap. Structural relaxations, charge densities, electron localization functions (ELFs), density of states (DOSs), band structures, and band decomposed charge density were obtained to reveal the amphoteric behavior of Bi in Hg_0.75_Cd_0.25_Te. The bonding characteristics between Bi and host atoms were discussed by analyzing charge densities and ELFs. The influence of Bi impurity on the electronic structure of Bi-doped Hg_0.75_Cd_0.25_Te was also analyzed by the calculated DOSs, band structures, and the band decomposed charge density of the defect band. It has been demonstrated that Bi can show a typical amphoteric substitution effect of group V elements.

## 1. Introduction

The infrared technique is widely used in the fields of military, medical treatment, metallurgy, chemical industry, aerospace, etc. [1]. Various materials, such as lead sulphide, indium arsenide, lithium tantalite, and HgCdTe, have been applied to the manufacture of infrared detection [2,3]. Compared with other materials, HgCdTe has the advantage of lower concentration of the intrinsic carrier, lower surface state density, smaller dielectric constant, and larger optical absorption coefficient [4]. As is known to all, the applications of semiconductor materials are mainly limited by the difficulty of n- or p-type doping via the incorporation of suitable impurities. N-type doping of HgCdTe is achieved easily by indium in situ substituting mercury, whose carrier concentration can be achieved above 10^18^ cm^−3^ [5]. However, well-controlled p-type doping is still one of the most serious bottlenecks in Hg_0.75_Cd_0.25_Te (MCT) based detector technology to date. The undoped p-type MCT is dominated by the presence of mercury vacancies, which acts as a trap center limiting the minority-carrier lifetime [6,7,8]. Hence, there has been an increased focus on replacing native acceptor defect doping with external acceptor dopants. The experimental results have indicated that the group V elements can provide a shallow acceptor level in HgCdTe. Meanwhile, the amphoteric behavior of the group V elements in MCT has been found in materials grown by bulk method, liquid phase epitaxy (LPE), and molecular beam epitaxy (MBE) [9,10,11]. It has been validated that the group V element Bi is an excellent p-type dopant in MCT grown by liquid phased epitaxy (LPE) from Te-rich solutions, and Bi-doped Hg_1−x_Cd_x_Te is amphoteric in epitaxially grown Te (Hg/Cd) [12,13]. However, the effects of Bi-doping on the electronic structure of Hg_1−x_Cd_x_Te still need further investigation, especially the bonding mechanism and band structure. Therefore, it is essential to have more comprehensive investigations on the fundamental physical properties of Bi-doped Hg_1−x_Cd_x_Te.

Density functional theory (DFT) is often used to calculate electronic structure. However, the choice of employing the local density approximation (LDA) or the generalized gradient approximation (GGA) often severely underestimates the band gap. Therefore, it is beneficial to apply a hybrid functional to correct the band gap underestimation in the first-principles calculations [14,15,16]. In this paper, we systematically perform the first-principles calculations based on DFT as implemented in the Vienna ab initio simulation package (VASP) code with a modified HSE hybrid exchange-correlation functional, band structures, and density of states. In addition, the ELF and the charge density of undoped Hg_0.75_Cd_0.25_Te and Bi-doped Hg_0.75_Cd_0.25_Te are also studied.

## 2. Results and Discussion

### 2.1. Atomic Relaxations

The existence of Bi impurity in Hg_0.75_Cd_0.25_Te induces structural relaxations of host atoms and modifies the electronic structure of the system. The relaxation results of the in situ impurity Bi_Te_ and the in situ impurity Bi_Hg_ in Hg_0.75_Cd_0.25_Te are listed in Table 1. The positive and negative signs denote outward relaxation and inward relaxation, respectively. The subscript is the number of the atomic shells around Bi. As is shown in Table 1, Hg^1^ cations around the in situ impurity Bi_Te_ show 0.68% relax inward. In contrast, the Cd^1^ cations around the in situ impurity Bi_Te_ show inward relaxation, about 0.11%. The inward relaxation between Hg^1^ and Cd^1^ is also shown in Table 1. Additionally, the bond angle of Hg-Bi-Cd (109.61°) increases while the Hg-Bi-Hg (109.33°) decreases for Hg_0.75_Cd_0.25_Te with Bi_Te_. The relaxation of the bond angles around the impurity Bi indicates that the inhomogeneous cation configuration makes the relaxations of Hg_0.75_Cd_0.25_Te with Bi_Te_, which produces an inverse change of the band angles Hg-Bi-Cd and Hg-Bi-Hg. The inward relaxation between Hg^1^ and Cd^1^ is caused by the bond angle relaxation. On the other hand, the covalent radii difference between dopant cation Bi and host atoms Te (the covalent radii are 1.46 Å and 1.36 Å for Bi and Te, respectively) causes the relaxations of the bond lengths.

**Figure 1:**
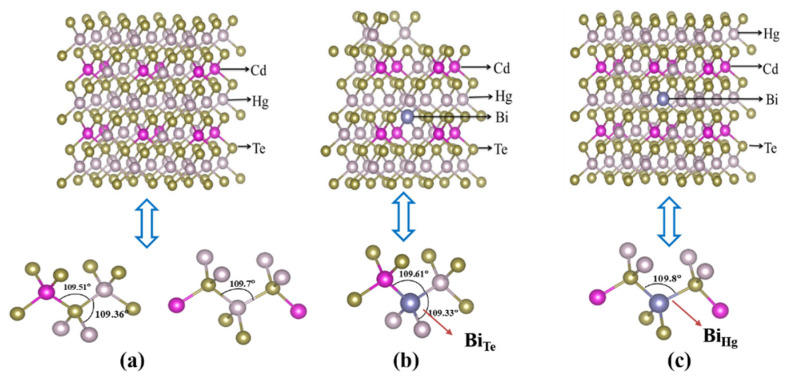
The 2 × 2 × 2 supercell for (**a**) undoped Hg_0.75_Cd_0.25_Te, (**b**) the in situ impurity Bi_Te_ in Hg_0.75_Cd_0.25_Te, and (**c**) the in situ impurity Bi_Hg_ in Hg_0.75_Cd_0.25_Te.

The outward relaxations of Hg_0.75_Cd_0.25_Te with Bi_Hg_ are also shown in Table 1. Te^1^ anions around the in situ impurity Bi_Hg_ show 6.31% relax outward, which is far greater than that of Hg_2_ (Cd_2_) cations. For Hg_0.75_Cd_0.25_Te with Bi_Hg_, the Te^1^-Hg^2^/Cd^2^ bond length is longer than that of Hg_0.75_Cd_0.25_Te. In Figure 1b, the bond angle of Hg-Bi-Cd is 109.61°. By contrast, the bond angle of Te-Bi-Te (109.8°) increases for Hg_0.75_Cd_0.25_Te with Bi_Hg_ in Figure 1c. The relaxation of the bond angles indicates that the inhomogeneous cation configuration makes the relaxations of Hg_0.75_Cd_0.25_Te with Bi_Hg_, which produces an inverse change of the bond angles.

### 2.2. Bonding Mechanism

To understand the bonding mechanism of Bi-doped Hg_0.75_Cd_0.25_Te, the valance charge density and ELFs [17,18] have been calculated. The charge density is a useful tool to describe the distribution of the electrons along with the bonding process.

**Figure 2:**
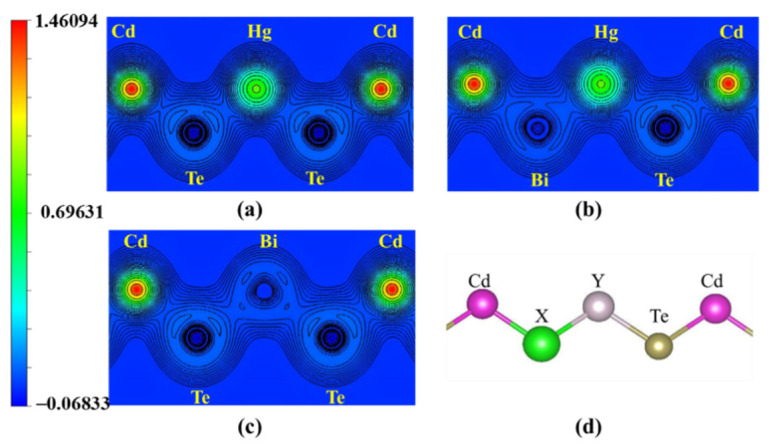
The valence charge density (2D) in the (1, 0, −1) plane for (**a**) undoped Hg_0.75_Cd_0.25_Te, (**b**) the in situ impurity Bi_Te_ in Hg_0.75_Cd_0.25_Te, and (**c**) the in situ impurity Bi_Hg_ in Hg_0.75_Cd_0.25_Te. (**d**) Local structure around Bi impurity.

The valence charge density in the (1, 0, −1) plane is shown in Figure 2. As shown in Figure 2b, the electrons accumulate along Bi–Hg/Cd bond, which shows strong covalent characteristics of Bi–Hg/Cd bonds and indicates a relatively stable structure after Bi in situ substituting Te. Comparing Figure 2a with Figure 2b, the electrons accumulation along Bi-Hg/Cd bond is weaker than that along Te-Hg/Cd, indicating the weaker bonding effect between Bi and Hg/Cd. As shown in Figure 2c, the charge distribution of Bi-Te is weaker than that of Hg-Te. Thus, we can project that Bi-Te bonds are greater and stronger than the substituted Hg-Te bonds.

**Figure 3:**
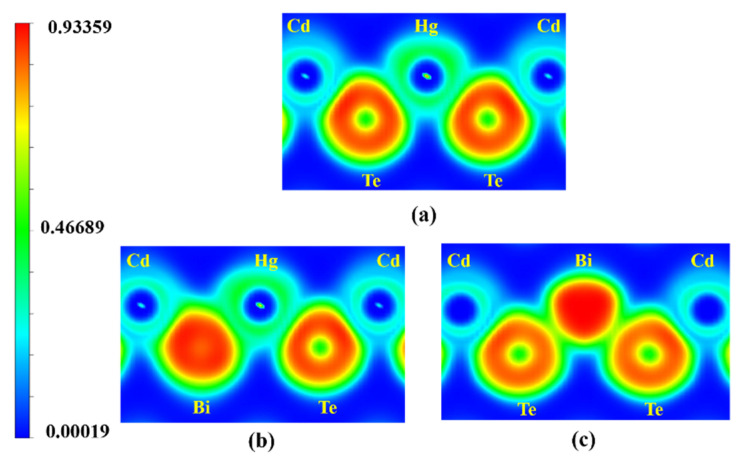
ELF in the (1, 0, −1) plane for (**a**) undoped Hg_0.75_Cd_0.25_Te, (**b**) the in situ impurity Bi_Te_ in Hg_0.75_Cd_0.25_Te, and (**c**) the in situ impurity Bi_Hg_ in Hg_0.75_Cd_0.25_Te.

As shown in Figure 3, the ELFs are calculated to analyze the bonding characteristics [17,19]. In Figure 3b, the value of ELF between Bi and Hg (Cd) is between 0.8–0.85, indicating the polar covalent bonding between them. In addition, the Te-Hg bond and Te-Cd bond also behave as the ELF, a polar covalent bonding in which ELF values around Hg atoms and Cd atoms are 0.85 and 0.87, respectively. In Figure 3c, for the in situ impurity Bi_Hg_ in Hg_0.75_Cd_0.25_Te, the ELF value around Bi atoms and nearest-neighbor Te atoms is 0.933 and 0.83, corresponding to strong covalent bonds. On the other hand, the ELF value of Hg-Te is about 0.87, implying the polar covalent bonding between Hg and Te. According to the results, the bonding characteristics of Bi-Hg (Cd) are similar to that of Te-Hg (Cd); the bonding characteristic of Bi_Te/Hg_- doped Hg_0.75_Cd_0.25_Te is similar to that of undoped-Hg_0.75_Cd_0.25_Te. Furthermore, a similar bonding characteristic indicates a relatively stable structure after Bi in situ substituting Te/Hg in Hg_0.75_Cd_0.25_Te.

### 2.3. Electronic Properties

**Figure 4:**
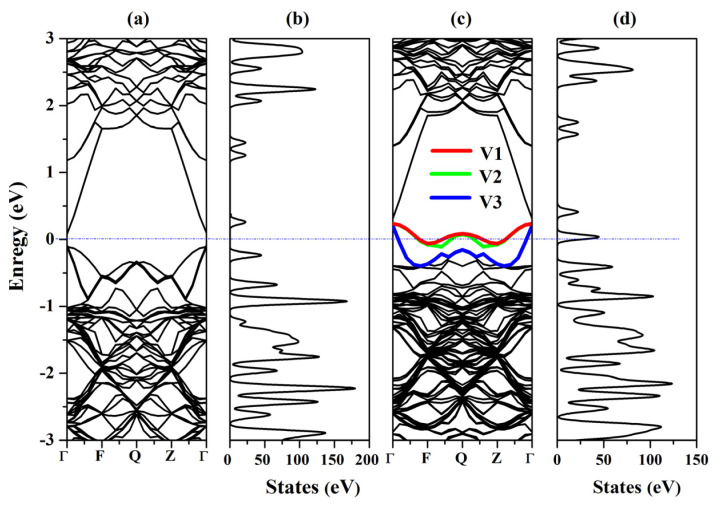
(**a**) Band structure of undoped Hg_0.75_Cd_0.25_Te, (**b**) total DOS of undoped Hg_0.75_Cd_0.25_Te, (**c**) band structure of Bi_Te_-doped Hg_0.75_Cd_0.25_Te, and (**d**) total DOS of the Bi_Te_-doped Hg_0.75_Cd_0.25_Te.

The band structure and DOS of undoped Hg_0.75_Cd_0.25_Te and Bi_Te_-doped Hg_0.75_Cd_0.25_Te are presented in Figure 4. Fermi level is set to zero. The conduction band minimum and valence band maximum in Figure 4a,c are both located at Г point, which illustrates that undoped Hg_0.75_Cd_0.25_Te and Bi_Te_-doped Hg_0.75_Cd_0.25_Te have direct band gaps. It can be obtained that the band gap of Hg_0.75_Cd_0.25_Te is 0.163 eV, which is in good agreement with the experimental value 0.166 eV [19]. Compared with Hg_0.75_Cd_0.25_Te, the band gap of Bi_Te_-doped Hg_0.75_Cd_0.25_Te in Figure 4c reduced significantly. It can be seen from Figure 4a,c that the band structure of Bi_Te_-doped Hg_0.75_Cd_0.25_Te is more intensive than that of Hg_0.75_Cd_0.25_Te. From Figure 4c, three defect bands emerge upon the top of the valence band (V1, V2, and V3), and the Fermi level shifts downward into the valence band. The in situ Bi_Te_ is the dominant acceptor.

**Figure 5:**
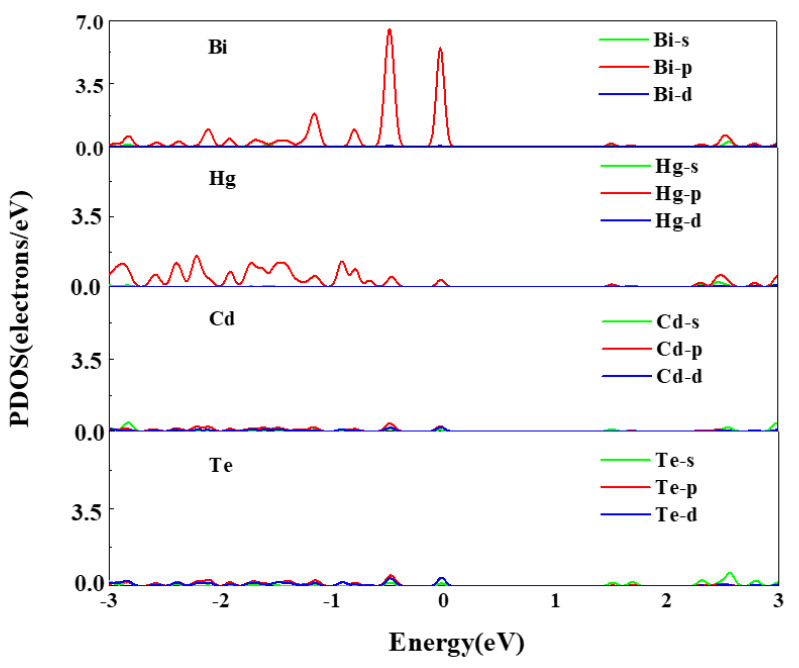
Partial DOSs of Bi, Hg, Cd, and Te in Bi_Te_-doped Hg_0.75_Cd_0.25_Te.

**Figure 6:**
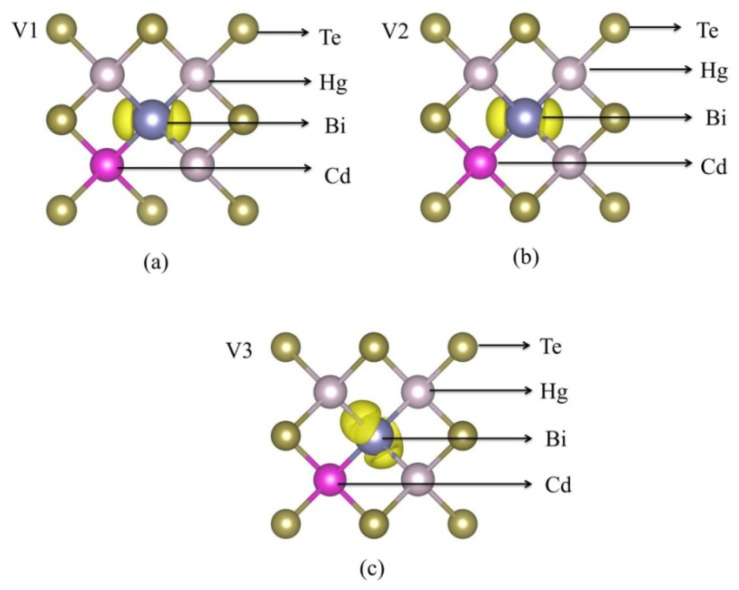
The band decomposed charge density of (**a**) V1, (**b**) V2, (**c**) V3, isosurfaces correspond to 0.003 e/Å.

As shown in Figure 5, the origin of these impurity states can be explored using the partial density of states (PDOS). Figure 5 illustrates that the states around the Fermi level mainly derive from Bi-p state after Bi in situ substituting Te. The p state and d state of Cd, the p state and d state of Te, and the p state of Hg forms relatively weaker peaks than Bi-p state near the VBM which can be ignored. To further investigate the composition of the VBM state, we calculate the band decomposed charge density of the three defect bands (V1, V2, and V3), as shown in Figure 6. The defect states at the Г points, from the V1 defect state to the V3 defect state in Figure 4, are described in Figure 6. We observed that the charge density is clearly localized at the impurity Bi atom. The charge transfer Bi, surrounding Hg and Cd atoms produce the defects states, leading to the reduction in the Hg_0.75_Cd_0.25_Te band gap. These isosurfaces for the charge density affirm that the defect states (V1, V2, and V3) originate from the Bi 6p orbit.

**Figure 7:**
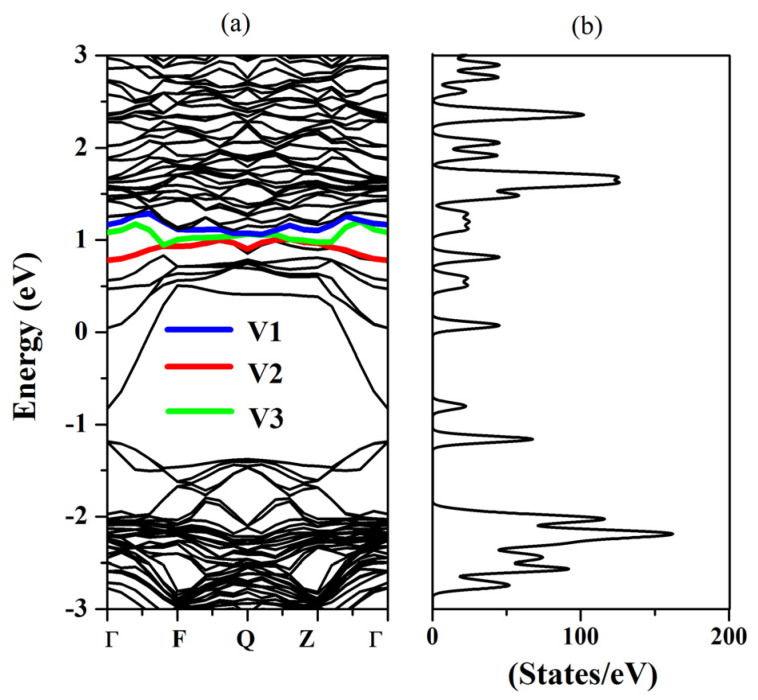
(**a**) The band structures and (**b**) total DOS of Bi_Hg_-doped Hg_0.75_Cd_0.25_Te.

The electronic properties of Bi_Hg_-doped Hg_0.75_Cd_0.25_Te have also been investigated. Figure 7 shows the band structure and DOS of Bi_Hg_-doped Hg_0.75_Cd_0.25_Te. Fermi level is set to zero. From Figure 7, the conduction band minimum and valence band maximum are both located at Г point, which indicates that Bi_Hg_-doped Hg_0.75_Cd_0.25_Te has a direct band gap. Compared with Hg_0.75_Cd_0.25_Te in Figure 4a, the band gap of Bi_Hg_-doped Hg_0.75_Cd_0.25_Te in Figure 7 reduces significantly. The impurity bands related to the doped Bi_Hg_ are highlighted. It is seen that the three levels of the conduction-band-maximum (CBM) at the Г point appeared separated, which is different from Bi_Te_-doped Hg_0.75_Cd_0.25_Te in Figure 4c. The Fermi level shifts upward into the conduction band, thus, Bi_Hg_-doped Hg_0.75_Cd_0.25_Te behave as a metal same with Hg_Bi_-doped Hg_0.75_Cd_0.25_Te. The in situ Bi_Hg_ is the dominant donor.

**Figure 8:**
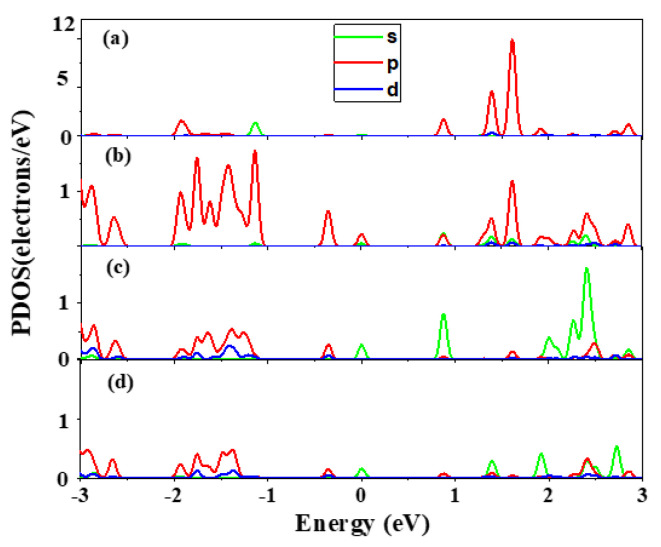
Partial DOSs of (**a**) Bi, (**b**) Te, (**c**) Hg, and (**d**) Cd in Bi_Hg_-doped Hg_0.75_Cd_0.25_Te.

**Figure 9:**
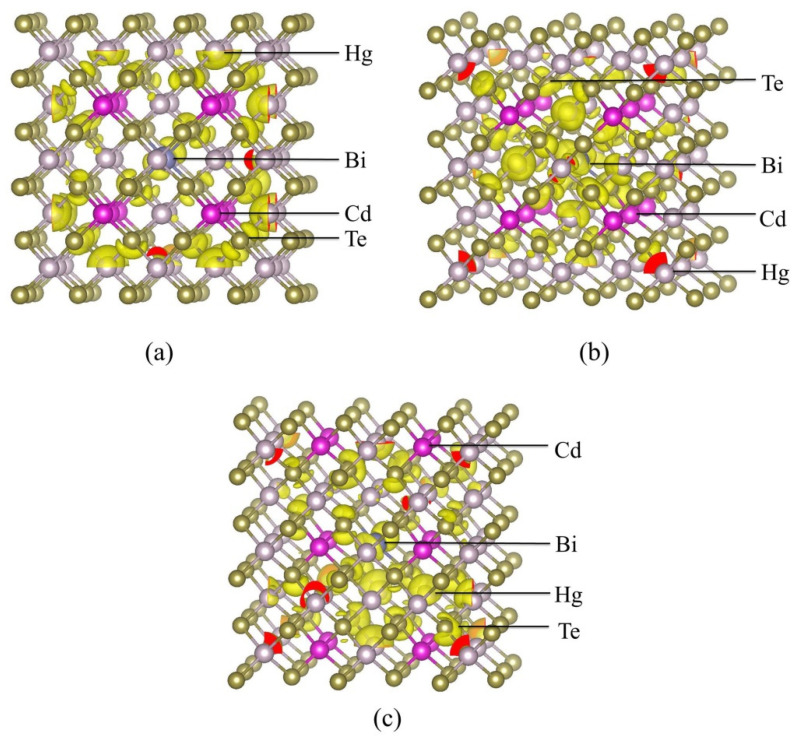
The band decomposed charge density of (**a**) V1, (**b**) V2, (**c**) V3, isosurfaces correspond to 0.006 e/Å.

We also calculated the partial density of states (PDOS) to study the origin of these impurity states. As shown in Figure 8, the defect states mainly arise from the Bi-p state, Te-p state, and Hg-s state after Bi in situ substituting Hg. The p state and d state of Cd, p state and d state of Te, and p state of Hg form an extremely weak peak, which can be ignored. Furthermore, the band decomposed charge density calculation for the defect states is performed, as shown in Figure 9. Figure 9 presents the charge distribution at the Г points of the Bi_Hg_-doped Hg_0.75_Cd_0.25_Te, from the V1 defect state to V3 in Figure 7. The intensity maximizes at the Bi site, but it is also present at the Te site and Hg site. From the configuration of the electronic state, we can also visualize that the defect band originates from the hybridization of Bi-p, Hg-s, and Te-p orbitals.

In this study, the two defect structures caused by Bi substitution have shown a certain degree of stability. In the HgCdTe system, Bi can show a typical amphoteric substitution effect of group V elements. In the Bi-Te defect system, Bi replaces the acceptor, which is represented as a P-type substitution, and in the Bi-Hg defect system, Bi replaces the donor, which is represented as an N-type substitution. These experiments prove that the mercury tellurium substituted by Bi has a certain degree of stability and effectiveness and therefore has a potential prospect in the doping effects on the electronic structure of Hg_1−x_Cd_x_Te.

## 3. Materials and Methods

Hg_1−x_Cd_x_Te is a pseudobinary alloy, which means two types of cations occupy the cation sites randomly. In order to simplify the calculation procedure, we chose the perfect quasi-zinc-blende crystal structure as the basis of the primitive cell. Through comparative analysis, it is found that the size of the supercell has a greater influence on the convergence speed of the calculation. The 2 × 2 × 2-supercell is sufficient to reflect the doping properties of HgCdTe, and is a more common choice [6,20]. Thus, we consider a 2 × 2 × 2-supercell with a total of 64 atoms, consisting of eight quasi-zinc-blende crystal structure of the unit cells of Hg_0.75_Cd_0.25_Te. As is shown in Figure 1a, each unit cell contains eight atoms, including four Te atoms, three Cd, and one Hg. In the first doping situation, the Te (0.375, 0.5, 0.625) atom was replaced by Bi atom as is shown in Figure 1b. In the second doping situation, the Hg (0.5, 0.5, 0.5) atom was replaced by Bi atom as is shown in Figure 1c. Our calculated results have validated that the dopant positions have an insignificant influence on the lattice parameters. By the well-known Vegard’s law, the relationship between lattice parameters and Cd composition of MCT can be expressed as [21]:

a = 6.46136 + 0.01999χ(1)

χ represents the component percentage of Cd in HgCdTe, and a represents the lattice parameter of a single HgCdTe, in nanometers.

The lattice parameters of Hg_0.75_Cd_0__.25_Te are 6.47. All calculations were performed by using VASP with projector-augmented wave (PAW) for the interaction between electrons and ions, and Perdew–Burke–Ernzerhof (PBE)-based HSE functional for the exchange-correlation. The cutoff energy for the plane-wave expansion was set to 500 eV. Hybrid functional mixed about 17% nonlocal Hartree–Fock exchange with 83% semilocal exchange, and the HSE screening parameter was set to a value of 0.2 Å^−1^. Brillouin-zone integration is performed on the Monkhorst–Pack scheme with a 2 × 2 × 2 mesh. A slight Gaussian broadening (σ = 0.05 eV) was applied so that the peaks of the defect would not merge with the band continuum for the calculations of the density of states (DOS). In the band structure calculations, k-point meshes were replaced by high symmetry point, which was set manually according to the Brillouin zone path. In addition, all structures were fully optimized until the force on each atom was smaller than 0.03 eV/Å. All the results were obtained based on the convergences.

## 4. Conclusions

In this study, the effects of Bi impurity on structural and electronic properties of Hg_0.75_Cd_0.25_Te have been systematically studied based on the first-principles calculations. The main conclusions can be drawn as follows:

- The covalent radii difference between dopant cation Bi and host atoms (Te and Hg) causes the relaxations of both bond lengths and bond angles;
- The results of charge density and ELFs indicate that the Bi impurity maintains relatively strong bonding characteristics with the host atom in Hg_0.75_Cd_0.25_Te;
- The impurity Bi shows a complicated amphoteric behavior in Hg_0.75_Cd_0.25_Te, which in situ substitutes the cation Hg behavior as n-type and in situ substitutes the anion Te behavior as p-type Hg_0.75_Cd_0.25_Te.

## Figures and Tables

**Table 1:** The relaxation results of Bi-doped Hg_0.75_Cd_0.25_Te.

Defect	Bond	Before (Å)	After (Å)	Change (Å)	Change Ratio (Å)
Bi_Te_	Bi-Hg^1^	2.802	2.783 (0)	−0.019	−0.68%
	Bi-Cd^1^	2.794 (0)	2.797 (0)	+0.003	+0.11%
	Hg^1^-Cd^1^	4.572	4.559	−0.013	−0.29%
Bi_Hg_	Te^1^-Bi	2.802	2.991	+0.189	+6.31%
	Cd^2^-Bi	4.572	4.632	+0.06	+0.13%
	Hg^2^-Bi	4.572	4.640	+0.068	+0.15%
	Te^1^-Hg^2^	2.802	2.833	+0.031	+1.1%
	Te^1^-Cd^2^	2.794	2.810	+0.015	+0.05%

## Data Availability

All data presented in this study are available upon request from the corresponding authors.

## Sample Availability

Samples of the compounds are available from the authors.
